# PMDFI: Predicting miRNA–Disease Associations Based on High-Order Feature Interaction

**DOI:** 10.3389/fgene.2021.656107

**Published:** 2021-04-09

**Authors:** Mingyan Tang, Chenzhe Liu, Dayun Liu, Junyi Liu, Jiaqi Liu, Lei Deng

**Affiliations:** School of Computer Science and Engineering, Central South University, Changsha, China

**Keywords:** miRNA-disease associations, high-order features, feature interactions, random forest, logistic regression

## Abstract

MicroRNAs (miRNAs) are non-coding RNA molecules that make a significant contribution to diverse biological processes, and their mutations and dysregulations are closely related to the occurrence, development, and treatment of human diseases. Therefore, identification of potential miRNA–disease associations contributes to elucidating the pathogenesis of tumorigenesis and seeking the effective treatment method for diseases. Due to the expensive cost of traditional biological experiments of determining associations between miRNAs and diseases, increasing numbers of effective computational models are being used to compensate for this limitation. In this study, we propose a novel computational method, named PMDFI, which is an ensemble learning method to predict potential miRNA–disease associations based on high-order feature interactions. We initially use a stacked autoencoder to extract meaningful high-order features from the original similarity matrix, and then perform feature interactive learning, and finally utilize an integrated model composed of multiple random forests and logistic regression to make comprehensive predictions. The experimental results illustrate that PMDFI achieves excellent performance in predicting potential miRNA–disease associations, with the average area under the ROC curve scores of 0.9404 and 0.9415 in 5-fold and 10-fold cross-validation, respectively.

## 1. Introduction

MiRNAs are short non-coding RNAs with length about 19–25 nucleotides (Ambros, 2001, 2004; Bartel, 2004). Since the first miRNA (lin-4) was discovered by Victor Ambros in 1993 (Lee et al., 1993), miRNA has been the most widely studied class of non-coding RNAs now (Esteller, 2011). Besides, it has been confirmed that miRNAs commonly exist in plants, animals, viruses, and human beings, and have an essential effect on cell growth, differentiation, and apoptosis because of its post-transcriptionally gene regulation by affecting the translation of mRNAs (Wienholds and Plasterk, 2005; Das et al., 2014; Zhao et al., 2017). The important influence of miRNAs on biological processes is manifested in most intronic miRNAs sharing promoter regions with host genes (Zhao et al., 2015). Therefore, it is natural for scientists to link miRNAs with human diseases and use them as biomarkers in the treatment of diseases. For example, miR-164a is highly expressed in pediatric acute lymphoblastic leukemia and pediatric acute myeloid leukemia (Zhang et al., 2009; Li et al., 2010). Studies demonstrated that miR-21 plays a crucial role in a plethora of biological diseases including cancer, cardiovascular diseases, and inflammation (Kumarswamy et al., 2011). Guay and Regazzi (2015) and Horsham et al. (2015) observed that the deregulation of miR-7 expression can potentially affect the adaptive capacity of β cells, contributing to the development of diabetes. The model-based computational approach proposed by Wang et al. (2008) identified five transcription factors and 7 miRNAs to be potentially responsible for the level of androgen dependency. Although miRNAs are proved to have close relationship with human disorders, the traditional biological methods to detect the underlying association between miRNAs and diseases are laboratory based, costly, and time consuming. Therefore, it is urgent and essential to apply computational methods to solve this issue. Nowadays, many computational methods are proposed to predict the novel association between miRNAs and diseases, and they are mainly divided into two categories: one is based on the assumption that the functional similarity of miRNAs tends to relate to similar diseases, and the other is based on machine learning.

According to the hypothesis that the functionally related miRNAs have a positive relationship with corresponding diseases, Chen and Zhang (2013) presented three methods based on the microRNA similarity, phenotype similarity, and network consistency similarity obtained by both of the two above similarity values, which are named as MBSI, PBSI, and NetCBI, respectively. Among these methods, NetCBI is better than the others with area under the ROC curve (AUC) of 0.8066, which still needs to be improved. Li et al. (2017) provided DeepWalk method that utilizes similarities within a known miRNA–disease association bipartite network to predict the unidentified miRNA–disease association when biological information, such as miRNA functional similarity and disease semantic similarity is unavailable. Although this method could reach the highest AUC of 0.937, it is incapable to predict associations of new miRNA or diseases that do not exist in the known network. Shen et al. (2017) integrated miRNA functional similarity, disease semantic similarity, and known miRNA–disease association, and then employed collaborative matrix factorization to predict the unknown miRNA–disease association (CMFMDA). CMFMDA could predict undiscovered miRNAs and diseases without known associations, but it may bias to miRNAs with more verified associated diseases. Chen et al. (2016) developed WBSMDA to reveal the novel miRNA–disease associations by integrating confirmed miRNA–disease associations, miRNA functional similarity, disease semantic similarity, and Gaussian interaction profile (GIP) kernel similarity of diseases and miRNAs, and obtained an average AUC of 0.8031. Then, they further raised the AUC to 0.9035 with an original method called HAMDA (Chen et al., 2017), which employs the hybrid graph-based recommendation algorithm to uncover the unrecognized associations between miRNAs and diseases.

As for methods based on machine learning, Peng et al. (2019) proposed a learning-based model named MDA-CNN. The method generates a three-layer network, including miRNA similarity network, disease similarity network, and protein–protein interaction network, to extract features and integrates an autoencoder and a convolutional network to select features and predict miRNA–disease association, respectively. Although the highest AUC the MDA-CNN achieved is 0.8897, the method performs well at the miRNA-phenotype association prediction. Zheng et al. (2019) presented a model based on machine learning named MLMDA, which utilizes miRNA sequence information extracted by k-mer sparse matrix, combing with other similarities of diseases and miRNAs. Besides, the MLMDA adopts a deep autoencoder to glean more latent features and uses the random forest (RF) to predict novel miRNA–disease associations. Chen et al. (2019) developed a method called EDTMDA, which applies principal component analysis (PCA) to reducing the dimension of features and utilizes ensemble learning to gain ultimate scores between miRNAs and diseases. EDTMDA's AUC could reach 0.9309 in LOOCV, but the dependence on the known associations between miRNAs and diseases may lead to a preference for miRNAs that have more associated diseases. Jiang et al. (2013) proposed an SVM-based method to identify disease-related microRNAs, which can distinguish positive microRNA-disease associations from negative microRNA-disease associations. In 10-fold cross-validation procedure, this method achieved the AUC of up to 0.8884. Zhang et al. (2019) proposed an unsupervised deep learning method implemented by variational autoencoder. The method combines miRNA similarity and disease similarity with identified associations to get two spliced matrices as the input of variational autoencoder, and then obtains the association scores of miRNA and disease. The model is not affected by the dearth of negative samples, but is hard to interpret.

In conclusion, the aforementioned computational methods could predict the underlying miRNA–disease associations effectively, but each one still has its own limits. In this paper, we propose a novel method called PMDFI, which is an ensemble approach for miRNA–disease associations prediction based on feature interaction learning. Our model can be divided into four parts: data set collection and processing, high-level feature extraction, feature interaction, and an integrated learning model. In detail, we gather miRNA–disease associations from HMDD v2.0, and calculate miRNA functional similarity, disease semantic similarity, GIP kernel similarly for miRNA, and disease. Then, after using the stacked autoencoder to extract the high-order features, we send them to the feature interactive layer to gain cross features. Finally, we design an ensemble model combining multiple RFs and logistic regression to predict potential miRNA–disease associations. In the experimental results, PMDFI has achieved excellent performance in predicting potential miRNA–disease associations, with AUC of 0.9404 and 0.9415 under 5-fold and 10-fold cross-validation, respectively.

## 2. Materials and Methods

### 2.1. Datasets for MDA Prediction

The experimentally supported miRNA–disease associations come from HMDD v2.0, which is derived from Li et al.'s work (Li et al., 2014). HMDD v2.0, a manual collected database, is used to annotate in details the miRNA–disease associations from genetics, epigenetics, circulating miRNAs, and miRNA-target interactions. We gather 5430 miRNA–disease association pairs encompassing 495 miRNAs and 383 diseases from the HMDD v2.0. In order to represent the associations between miRNA *m*(*i*) and disease *d*(*j*), we construct an adjacency matrix A_495×383_, where element *A*(*i, j*) = 1 indicates that miRNA has a definite association with disease, and element *A*(*i, j*) = 0 indicates that the association between miRNA and disease is uncertain. Matrix *A* is a sparse matrix with 5,430 of “1,” i.e., 5,430 miRNA–disease association pairs, and we take these pairs as positive samples. As for the negative samples, according to Zhou et al. (2020), all “0”s (miRNA–disease pairs with no definite association) in the matrix *A* are divided into 23 clusters with k-means clustering, and the same amount of samples are randomly selected from each cluster to form 5,418 negative samples. It is worth noting that, in order to ensure the validity of comparative experiments, the positive and negative samples in our datasets are the same as Zhou et al.'s work.

### 2.2. MiRNA and Disease Information Profiles

#### 2.2.1. MiRNA Functional Similarity

The miRNA functional similarity is useful to predict the functions of unknown miRNAs and study the interactions between miRNAs, because miRNAs with similar functions tend to trigger pathologically similar diseases. The miRNA functional similarity matrix can be represented as follows:

(1) $$\begin{array}{r} FS={\left[m_{1},m_{2},\cdots \;,m_{nm}\right]}^{T},m_{i}\in {ℜ}^{km} \end{array}$$

where *nm* is the number of miRNAs and *km* is the size of the vector that represents an miRNA.

Here, we download miRNA function similarity between miRNA pairs directly from http://www.cuilab.cn/fles/images/cuilab/misim.zip, which calculated by Wang et al.'s work based on advanced MISIM method (Wang et al., 2010). The miRNA functional similarity matrix *FS* is a matrix with 495 rows and 495 columns, and element *FS*(*m*_*i*_, *m*_*j*_) represents the functional similarity between *miRNA*(*i*) and *miRNA*(*j*).

#### 2.2.2. Disease Semantic Similarity

If an miRNA has been proved to be linked to a certain disease, it is possible that the miRNA is also related to other diseases with similar phenotypes. Therefore, the semantic similarity of the disease is effective in large-scale research on the association between disease and miRNA. The disease semantic similarity is described as directed acyclic graph (DAG), and

(2) $$\begin{array}{r} DAG\left(d\right)=\left\{d,T\left(d\right),E\left(d\right)\right\} \end{array}$$

where *d* is the disease itself, *T*(*d*) is a set of nodes consisting of disease *D* and all its ancestor nodes, and *E*(*d*) corresponds to the edge set of the direct link from the parent node to the child node.

We collect disease semantic similarity from MeSH database (http://www.ncbi.nlm.nih.gov/), which has been widely adopted to study miRNA–disease associations (Zou et al., 2016). And each disease in DAG can be calculated as follows:

(3) $$\{\begin{array}{l} D1_{D}(d)=1\;if\;d=D\\ D1_{D}(d)=\max\;\left\{0.5\times D1_{D}\left(d^{\prime }\right)|d^{\prime }\in \text{ child of }d\right\}\;if\;d\neq D \end{array}$$

and

(4) $$\begin{array}{r} DV\left(D\right)=\underset{d\text{∈}T\left(d\right)}{\sum }D_{D}\left(d\right) \end{array}$$

Then the semantic similarity score between *diseases*(*i*) and *diseases*(*j*) is defined as follows:

(5) $$SS(d(i),d(j))=\frac{\sum_{t\in T(d(i))\cap T\left(d_{0j}\right)}\left(D_{d(i)}(t)+D_{d(j)}(t)\right)}{DV(d(i))+DV(D(j))}\text{​}.$$

#### 2.2.3. GIP Kernel Similarly for miRNA and Disease

GIP kernel similarity originates from the topological structure of the known interaction network, which is beneficial for predicting the miRNA–disease associations (Wang et al., 2010). We adopt a binary vector *IP*(*d*), a row in the adjacency matrix, to express the interaction profile of disease d with each miRNA, and the disease GIP kernel similarity between disease *d*(*i*) and *d*(*j*) can be calculated as follows:

(6) $$\begin{array}{r} GS_{d}\left(d_{i},d_{j}\right)=\text{exp }\left(-\gamma_{d}\|IP\left(d_{i}\right)-IP\left(d_{j}\right){\|}^{2}\right) \end{array}$$

and

(7) $$\begin{array}{r} \gamma_{d}=\lambda_{d}^{\prime }/\left(\frac{1}{n}\overset{n}{\underset{i=1}{\sum }}\|IP\left(d_{i}\right){\|}^{2}\right) \end{array}$$

where *n* is the number of human diseases and equals to 383, *γ*_*d*_ is an adjustable parameter of the kernel bandwidth, and $\lambda_{d}^{\prime }=1$ according to van Laarhoven et al.'s work (van Laarhoven et al., 2011). Similarly, we can use a binary vector IP(m) to express the interaction profile of miRNA m with each disease, and the GIP kernel similarly between miRNA *m*(*i*) and *m*(*j*) can be calculated as follows:

(8) $$\begin{array}{r} GS_{m}\left(m_{i},m_{j}\right)=\text{exp }\left(-\gamma_{m}\|IP\left(m_{i}\right)-IP\left(m_{j}\right){\|}^{2}\right) \end{array}$$

and

(9) $$\begin{array}{r} \gamma_{m}=\lambda_{m}^{\prime }/\left(\frac{1}{m}\overset{m}{\underset{i=1}{\sum }}\|IP\left(m_{i}\right){\|}^{2}\right) \end{array}$$

where *m* is the number of miRNAs and equals to 495, for the same reason, $\lambda_{m}^{\prime }$ is set to 1.

### 2.3. PMDFI Framework

**Figure 1:**
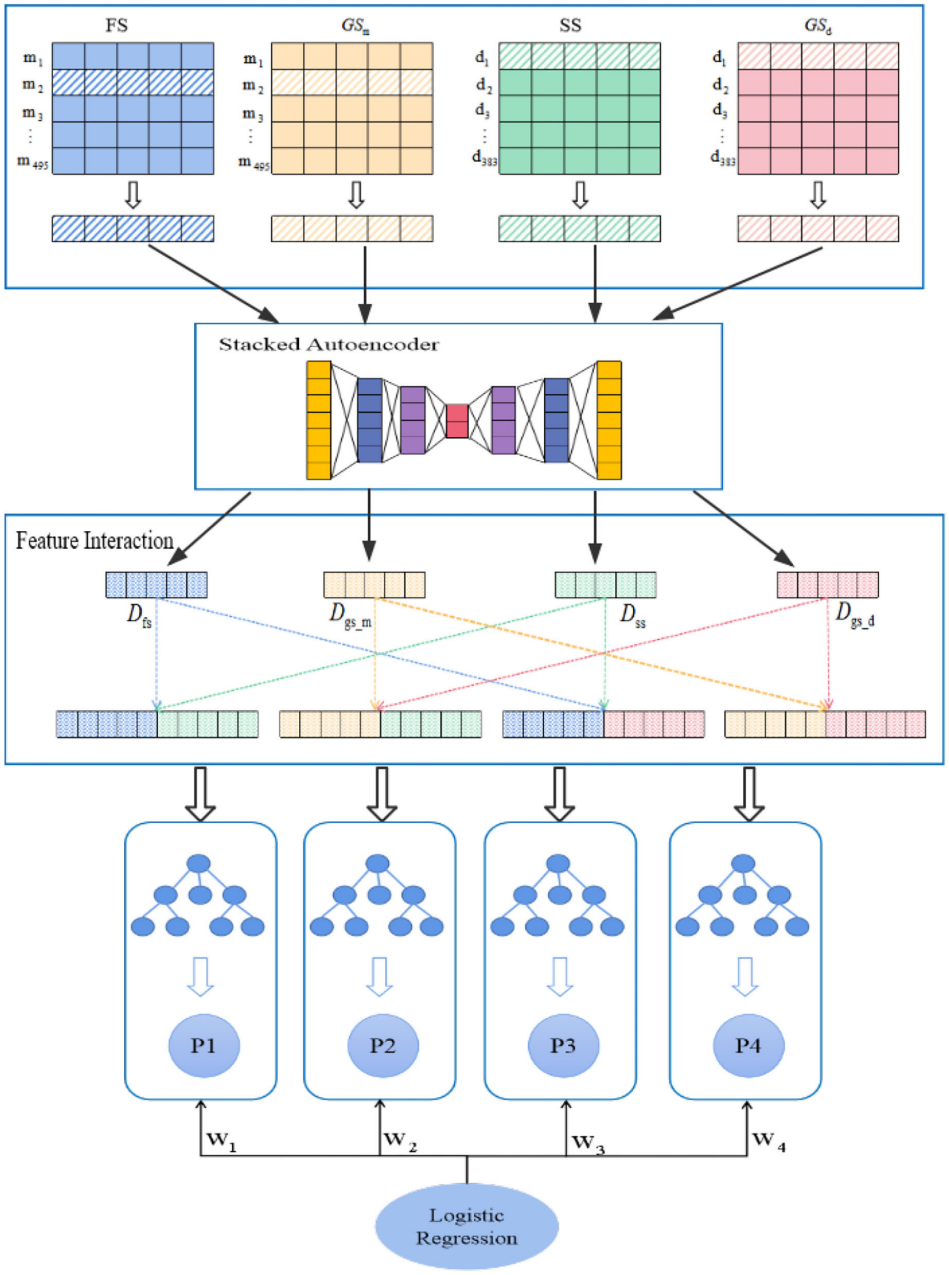
Flowchart of PMDFI model to predict potential microRNAs (miRNAs)–diseases associations. The model can be divided into four parts: data set collection and processing, high-order feature extraction, feature interaction, and an integrated learning model. First, we gather miRNA–disease associations from HMDD v2.0, and form the similarity matrix between miRNA and disease; second, we adopt a stacked autoencoders to extract high-order features; then, we use the interaction features layer to learn the interaction between different features. Finally, we combine multiple random forest (RF) with logistic regression to predict potential miRNA–disease associations.

In this study, we construct a model named PMDFI to predict potential miRNA–disease associations. The flowchart of PMDFI is shown in Figure 1. In the data set collection and processing stage, we gather 495 miRNAs and 383 diseases from the HMDD v2.0 database to form an adjacency matrix A_495×383_, including 5430 miRNA–disease pairs with definite associations. Then, we acquire miRNA functional similarity (*FS*), disease semantic similarity (*SS*), and GIP kernel similarity for miRNA (*GS*_*m*_) and disease (*GS*_*d*_). For each miRNA–disease pair, we extract four one-dimensional features, which include a 1 × 495 miRNA functional similarity feature, a 1×383 diseases semantic similarity feature, and a 1 × 495 and 1 × 383 GIP kernel similarity for miRNAs and disease. Then these features are input in parallel into the stacked autoencoder to extract high-order features, instead of directly concatenating and averaging them. In this way, our method has the ability to learn the internal deep connections in the feature matrix, which have been previously ignored due to the lack of miRNA functional similarity or diseases semantic similarity. In the feature interaction layer, the high-order features derived from stacked autoencoder are sent to perform feature interaction learning, which aims at obtaining four cross features containing the internal potential relationship of miRNA (disease) and the interaction information among those features. Finally, the obtained cross features are independently input into the four RF models for training, and a set of four prediction scores is calculated for each sample input. During each iteration, we constantly adjust the weight of each RF model, and adopt a logistic regression to make a final comprehensive prediction.

#### 2.3.1. Stacked Autoencoder to Extract High-Order Features

**Figure 2:**
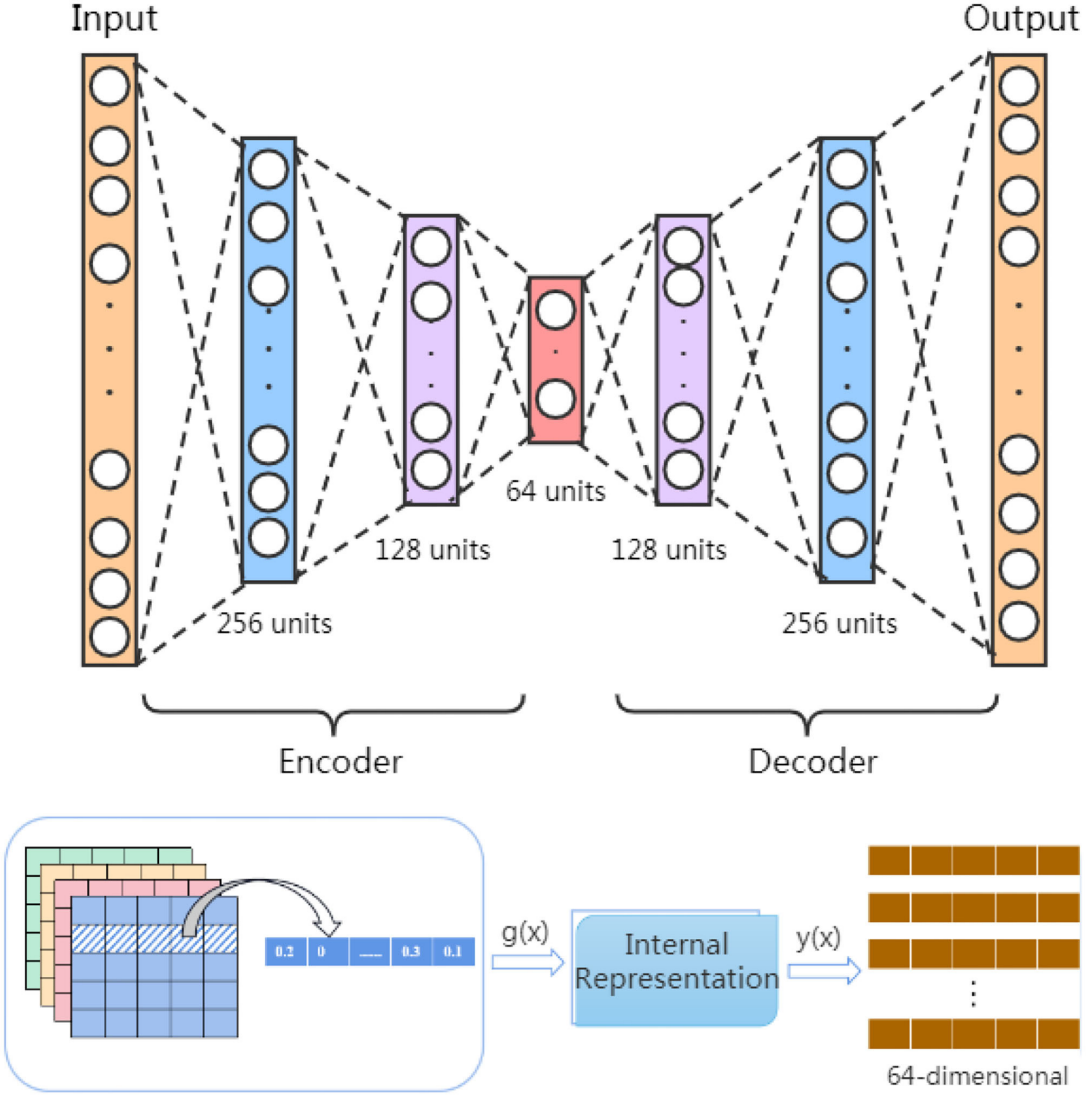
Extract high-order features based on autoencoder.

These four similarities matrix information (*FS*, *SS*,*GS*_*m*_, and *GS*_*d*_) have inevitable restriction that they are unable to present the inner deep connections among different miRNAs (diseases) due to low-order feature representations. To tackle this obstacle, inspired by Song et al.'s work (Song et al., 2019), we use a stacked autoencoder to extract meaningful high-order features for miRNA and disease from the established similarity network. The autoencoder is an artificial neural network that can learn the efficient representation of input data through unsupervised learning (Vincent et al., 2008; Shu et al., 2018). As a powerful feature detector, the autoencoder encodes the original input feature and reduces the dimensionality to find implicit associations between the input feature, and extracts expressive high-order features. As shown in Figure 2, the stacked encoder consists of two parts: an encoder (also known as the recognition network) and a decoder (also known as the generation network). The encoder converts the input feature into an internal representation, and the decoder converts the internal indicates conversion to output.

In order to learn high-order features, we build a stacked autoencoder that includes three hidden layers with 256, 128, and 64 units. The stacked autoencoder means that the feature vectors in the previous autoencoder are used as the input of the next autoencoder, and the whole training process is greedy in a layered manner. In our model, the feature information of *FS* = {fs_1_, *fs*_2_, ⋯ , *fs*_495_}, *SS* = {*SS*_1_, *SS*_2_, ⋯ , *SS*_495_},*GS*_*d*_ = {*d*_1_, *d*_2_, ⋯ , *d*_383_} and *GS*_*m*_ = {*m*_1_, *m*_2_, ⋯ , *m*_495_} is input into stacked autoencoder *H*1, *H*2, *H*3, and *H*4, respectively, and divided into four parallel groups for high-order feature extraction by minimizing the discrepancy between the input features and the reconstruction ones.

Initially, we set *N*_*L*_ and*N*_*G*_*i*__as the number of units in the input layer and the *i*th hidden layer, and use one feature vector $x\in R^{N_{L}\text{×}1}$ to represent those input feature vectors. Subsequently, during the encoding process, the autoencoder transforms *x* into a latent representation *g*^(*i*)^ through a composite mapping of linear transformation and non-linear activation function *f*, as shown in the following equation:

(10) $$\begin{array}{r} g^{\left(i\right)}=f\left(W_{1}^{\left(i\right)}x+b_{1}^{\left(i\right)}\right) \end{array}$$

where *i* is *i*th hidden layer, $g^{\left(i\right)}\in R^{N_{G_{i}}}$ is the latent feature, $W_{1}^{\left(i\right)}\in R^{N_{G_{i}}\text{×}N_{L}}$ is the encoding weight matrix, $b_{1}^{\left(i\right)}\in R^{N_{G_{i}}}$ is the bias vector, and *f* (·) is the sigmoid function.

Here, we adopt three hidden layers, i.e., *i* = 3. Then there is the process of decoding, which learns features inverse mapping. The latent representation *y*^(*i*)^ is mapped to a feature vector as follows:

(11) $$\begin{array}{r} y^{\left(i\right)}=f\left(W_{2}^{\left(i\right)}g^{\left(i\right)}+b_{2}^{\left(i\right)}\right) \end{array}$$

similarly, *g*^(*i*)^ is the latent data, $W_{2}^{\left(i\right)}\in R^{N_{L}\text{×}N_{G_{i}}}$ is the decoding weight matrix, $b_{2}^{\left(i\right)}$ is the bias vector.

Given a training feature vector *x*(*k*), which can be shown as: *x*(*k*) = {*f*_*S*_(*k*), *ss*(*k*), *d*(*k*), *m*(*k*) (*Denotedasχ* = {FS, SS, GS_*d*_, GS_*m*_}), we can learn the underlying features by minimizing the reconstruction error of the cost function:

(12) $$\begin{array}{r} H_{N}\left(X,Y,\theta \right)=\frac{1}{2}\overset{m}{\underset{K=1}{\sum }}\|x\left(k\right)-y\left(k\right){\|}_{2}^{2}+\lambda \|\theta {\|}_{2}^{2} \end{array}$$

where *N* = 1, 2, 3, 4, and *Y* represents all the reconstructed feature vectors, *y*(*k*) is the *k*th reconstructed feature vector, *x*(*k*) is the *k*th training feature vector, *m* is the number of training feature vectors, λ is the weight decay parameter, *θ* = {W, b}, *W* is the weight, and b is the biases of the autoencoder.

#### 2.3.2. Feature Interaction

In the previous section, we have obtained four different types of high-order features (*D*_*fs*_, *D*_*ss*_, *D*_g_s__−_*m*_, and *D*_g_s__−_*d*_) derived from miRNA functional similarity, disease semantic similarity, and GIP kernel similarity for miRNA and disease. However, these four features are unilateral feature representations, which only express the degree of closeness among different miRNAs (diseases) and extract their meaningful latent connections. An effective prediction accuracy not only depends on valuable high-order features, but also on the feature interactive information. Therefore, we obtain cross features by combining different high-order features and use them to learn feature interaction information.

In our model, a feature interaction layer is adopted to gain the interaction information between different high-order features. Considering the miRNA–disease associations, we combine the two features of miRNA with the two features of disease, respectively, and gain a total of four cross features. In order to predict the association between a specific miRNA and a certain disease, *D*_*fs*_ and *D*_*ss*_ are simultaneously mapped to the same space to obtain cross features, which can be expressed as:

(13) $$\begin{array}{r} D_{1}={\left[\begin{array}{c} D_{fS}\\ D_{SS} \end{array}\right]}^{T} \end{array}$$

Similarly, the other three cross features are shown as follows:

(14) $$D_{2}={\left[\begin{array}{l} D_{{\text{gs}}_{-}}m\\ D_{ss} \end{array}\right]}^{T}$$

(15) $$D_{3}={\left[\begin{array}{l} D_{\text{fs}}\\ D_{gs_{-}}d\; \end{array}\right]}^{T}$$

(16) D4=[g Ds_mdDgs_ ]T

As a result, the high-order features of miRNA and disease are mapped to different spaces for feature interaction, and four unilateral high-order features are converted into four cross features with deep interactivity.

#### 2.3.3. Ensemble Model Based on Multiple RF and Logistic Regression

An RF consists of an set of classification trees, and each tree divides the feature space into different regions based on the division of each node in the tree. During the training process, the randomness allows the trees to give independent estimates, which collectively contribute to achieve accurate and robust results. Here, we use four RFs and each RF is consisted of 300 independent trees. The core idea of our model is to input four interactive cross features into respective RF in parallel for self-learning and model building, and then merge the four RFs with logistic regression to make comprehensive predictions.

Our dataset includes 5,430 positive samples labeled as “1,” and 5,418 negative samples labeled as “0.” The input sample *x*_*k*_ of each four cross features covers diversified feature information and the four cross features could be represented as $f_{k}=\left\{D_{1}^{\left(k\right)},{\text{D}}_{2}^{\left(k\right)},{\text{D}}_{3}^{\left(k\right)},{\text{D}}_{4}^{\left(k\right)}\right\}$, $\left(D_{N}\in {ℜ}^{1\text{×}64},\;\left(N=1,2,3,4\right)\right)$. And we use θ_*R*_ = {[*x*_1_; f_1_], [*x*_2_; f_2_], ⋯ , [*x*_*m*_; f_*m*_]} to denote all training miRNA–disease pairs, where *m* is the number of all training sample pairs. In order to train a robust model, all samples are randomly input into the random forest for pre-training. For a sample *x*_*k*_, the interactive cross features *f*_*k*_ are input into the corresponding RF, and a set of prediction score can be obtained and expressed as, $p^{\left(k\right)}=\left\{p_{1}^{\left(k\right)},p_{2}^{\left(k\right)},p_{3}^{\left(k\right)},p_{4}^{\left(k\right)}\right\}$. $p_{N}^{\left(k\right)}$ is a probability score between 0 and 1, which represents the degree of association between a miRNA and a disease. Subsequently, we use logistic regression to do the final classification task for each miRNA–disease pair, instead of simply averaging the probability score of the four RF regression models. We consider the score *P*^(*k*)^ of each sample pair *x*_*k*_ as a new feature ${\text{x}}^{\prime \left(k\right)}=\left\{x_{1}^{\prime \left(k\right)},x_{2}^{\prime \left(k\right)},x_{3}^{\prime \left(k\right)},x_{4}^{\prime \left(k\right)}\right\}$ and assign it a weight $W^{\left(k\right)}=\left\{w_{1}^{\left(k\right)},w_{2}^{\left(k\right)},w_{3}^{\left(k\right)},w_{4}^{\left(k\right)}\right\}$, and constantly update the weights during each iteration. After logistic regression training, the comprehensive prediction performance can be expressed as: *Y* = *w*^*T*^*x*′ + *b*, where b is a constant. Finally, We conduct 5-fold cross-validation and 10-fold cross-validation on all samples to test the performance of our method.

## 3. Results and Discussion

### 3.1. Evaluation Criteria

To assess the performance of PMDFI, we adopt 5-fold cross-validation (5-CV) and 10-fold cross-validation (10-CV) as well as several widely used measures, including recall, precision, F1-score, AUC, and area under the PR curve (AUPR). And these measures are calculated as follows:

(17) $$\begin{array}{r} \text{Recall}=\frac{TP}{TP+FN} \end{array}$$

(18) $$\begin{array}{r} \text{Precision}=\frac{TP}{TP+FP} \end{array}$$

(19) $$\begin{array}{r} F1-\text{score}=\frac{2\times \text{Precision }\times \text{ Recall}}{\text{Precision }+\text{ Recall}} \end{array}$$

where *TP*, *FP*, *TN*, and *FN* represent the true positive, false positive, true negative, and false negative, respectively.

### 3.2. Prediction of miRNA–Disease Association Based on PMDFI

We use 5-fold and 10-fold cross-validation to evaluate the performance of PMDFI in predicting miRNA–disease associations. In 5-CV (10-CV), all sample pairs are randomly divided into five (10) equal groups, and four (nine) groups of them are regarded as training samples, and the remaining one group is used as test samples. Table 1 lists the results of 5-CV and 10-CV obtained by PMDFI, and indicates that under 5-CV (10-CV), the AUC, AUPR, Precision, Recall, and F1-score of PMDFI are 0.9404 (0.9415), 0.9373 (0.9385), 0.8663 (0.8669), 0.8812 (0.8832), and 0.8736 (0.8748), respectively. The average AUC of our model exceeds 0.94 in either the 5-fold cross-test or the 10-fold cross-test. Therefore, the results fully demonstrate that PMDFI has a good performance in predicting the latent associations between miRNAs and diseases.

**Table 1:** The results of 5-fold and 10-fold cross-validation obtained by PMDFI.

**C. val**.	**AUC**	**AUPR**	**Precision**	**Recall**	**F1-score**
5-CV	0.9404	0.9373	0.8663	0.8812	0.8736
10-CV	0.9415	0.9385	0.8669	0.8832	0.8748

### 3.3. Comparison With Existing State-of-the-Art Methods

**Figure 3:**
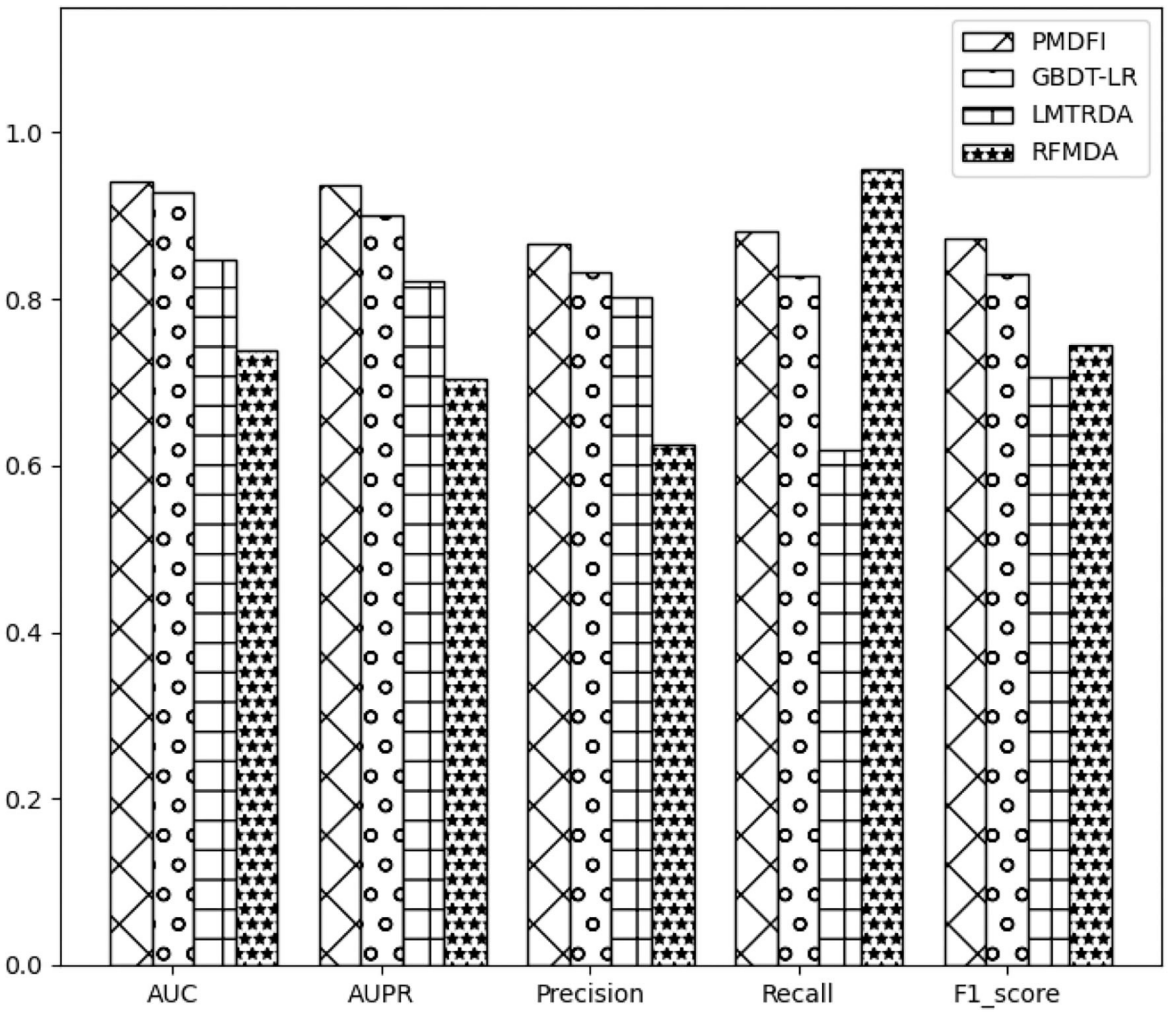
Histograms of the results of different methods based on 5-fold cross-validation.

In order to systematically evaluate the performance of PMDFI, we compare our method with other state-of-the-art computational models, such as GBDT-LR (Zhou et al., 2020), LMTRDA (Wang et al., 2019), and RFMDA (Chen et al., 2018). GBDT-LR is a original model that combines gradient boosting decision tree with logistic regression to prioritize miRNA candidates for diseases. LMTRDA is a logistic model tree used to predict miRNA–disease associations by fusing multi-source information. RFMDA is a computational model of random forest for miRNA–disease associations prediction based on machine learning. The comparison between PMDFI and these models is carried out based on 5-CV and illustrated specifically in Table 2. From the table, PMDFI, GBDT-LR, LMTRDA, and RFMDA models achieve AUC of 0.9404, 0.9274, 0.8479, and 0.7388, respectively, and PMDFI presents the best performance. PMDFI outperforms GBDT-LR by 1.3%, LMTRDA by 9.25%, and RFMDA by 20.16% in terms of AUC. Figure 3 further describes the comparison of our method with other methods in 5-CV with the format of histograms, and the leftmost one represents our method. In conclusion, except that the recall is 0.0736 lower than RFMDA, PMDFI makes a significant improvement in the field of prediction for potential miRNA–disease associations.

**Table 2:** The comparison of different methods based on 5-fold cross-validation.

**Method**	**AUC**	**AUPR**	**Precision**	**Recall**	**F1-score**
PMDFI	0.9404	0.9373	0.8663	0.8812	0.8736
GBDT-LR	0.9274	0.9014	0.8315	0.8273	0.8302
LMTRDA	0.8479	0.8217	0.8013	0.6190	0.7076
RFMDA	0.7388	0.7034	0.6253	0.9548	0.7453

### 3.4. Comparison With Different Interactive Cross Features

In order to further illustrate the contribution of distinct interactive cross features to the potential miRNA–disease associations prediction, we separately input cross features D1 (*D*_fs_ ⊕ *D*_*ss*_), D2 (*D*_fs_ ⊕ *D*_*g*_*s*__−_*d*_), D3 (*D*_g_s__−_m_ ⊕ *D*_*ss*_), and D4 (*D*_gs_m ⊕ *D*_*g*_*s*__−_*d*_) into the RF model for training, without integrating the overall performance of the four cross features. Table 3 displays the performance of each interactive cross features on miRNA–disease potential association prediction.

**Table 3:** Comparison of the performance of four interactive cross features.

**Method**	**AUC**	**AUPR**	**Precision**	**Recall**	**F1-score**
D1(*D*_fs_ ⊕ *D*_*ss*_)	0.9106	0.9093	0.8289	0.8388	0.8338
D2(*D*_fs_ ⊕ *D*_*g*_*s*__−_*d*_)	0.9283	0.9240	0.8513	0.8692	0.8601
D3(*D*_g_s__−_m_ ⊕ *D*_*ss*_)	0.9239	0.9193	0.8381	0.8642	0.8509
D4(*D*_gs_m ⊕ *D*_*g*_*s*__−_*d*_)	0.9392	0.9334	0.8630	0.8834	0.8730
PMDFI	0.9404	0.9373	0.8663	0.8812	0.8736

**Figure 4:**
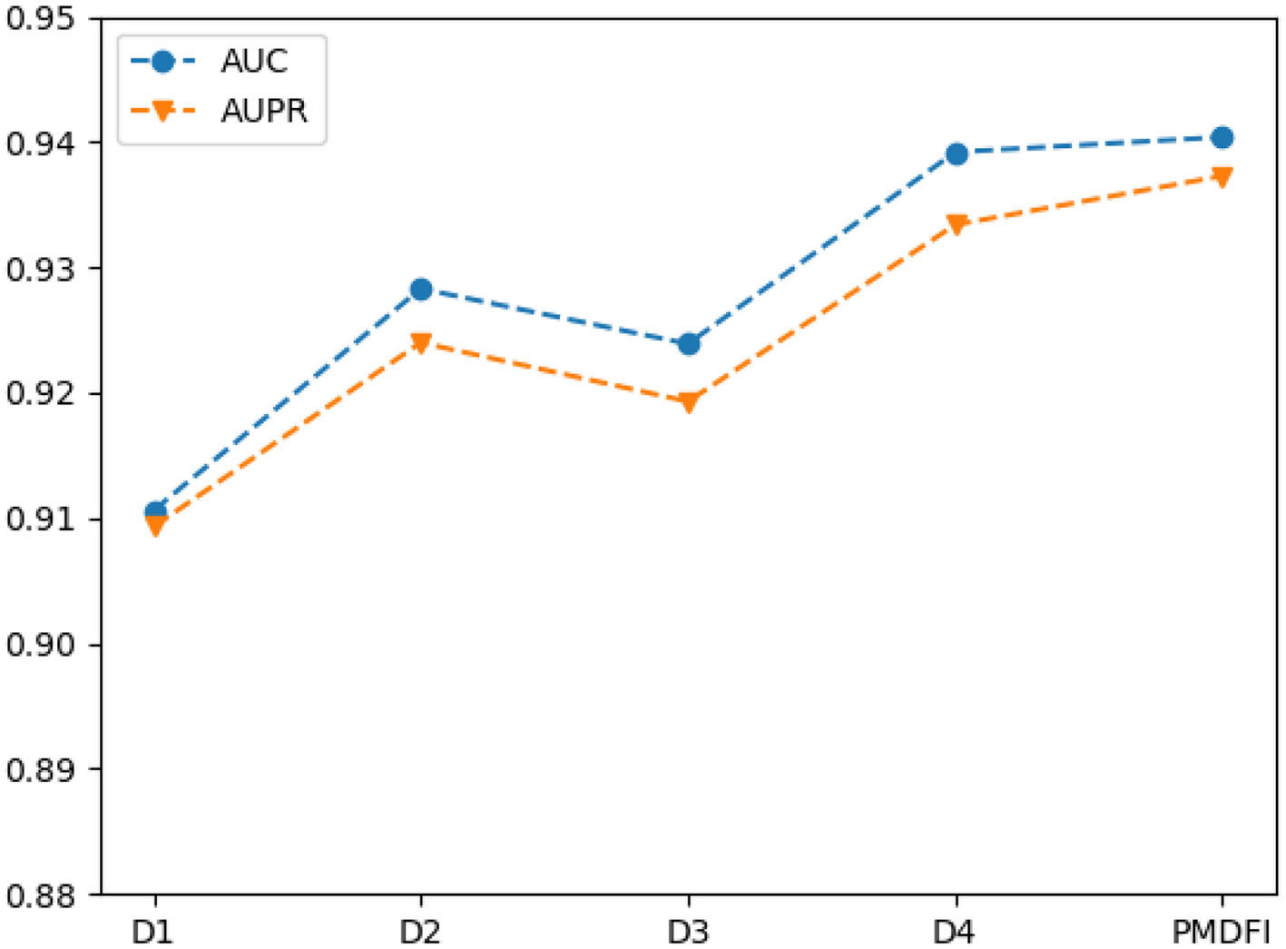
Line chart of area under the ROC curve (AUC) and area under the PR curve (AUPR) scores of different interaction cross features.

In the table, the AUC and AUPR score of the four interactive cross features fluctuate in the range of 0.9249 ± 0.0143 and 0.9213 ± 0.0121, respectively. And the cross feature *D*1 has the worst performance with an AUC of 0.9106, which is 2.98% lower than the optimal score. Besides, the D4 cross feature has the best performance compared to other three, and its AUC, AUPR, Precision, Recall, and F1-score are 0.9392, 0.9334, 0.8630, 0.8834, and 0.8730, respectively. Although *D*4 is the best performer among the four cross features, the performance of it is still slightly worse than that of the integration of the whole four features. For a clearer comparison, we also draw a line graph of the four interactive cross features and their combinations in terms of AUC and AUPR values. Figure 4 gives a clue that the performance of integrating the four interactive cross features is the best, and its AUC and AUPR values are both at the highest point.

### 3.5. Comparison With Different Classifier Models

**Figure 5:**
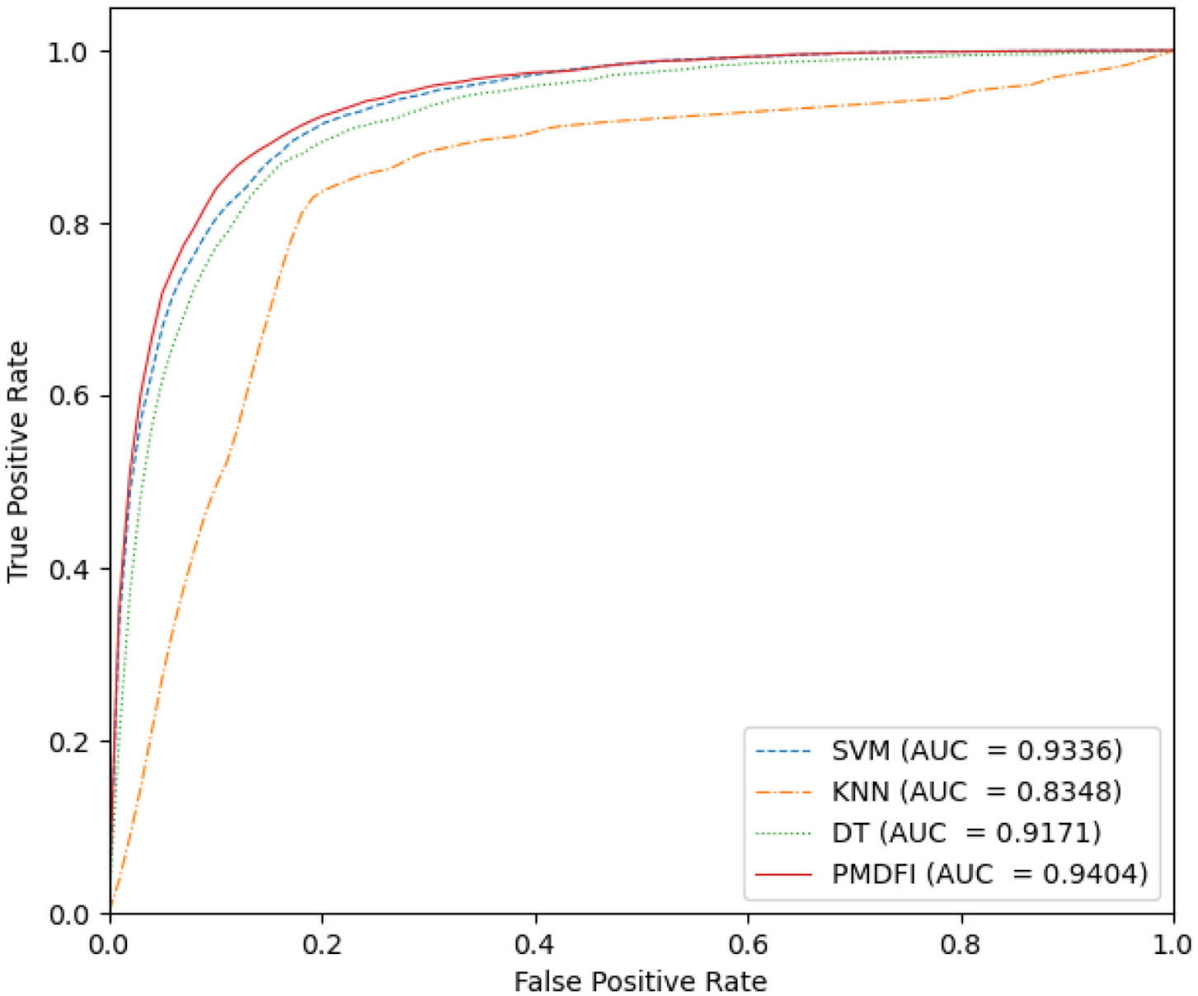
The ROC curves of different classifier models.

In our method, we use an ensemble learning model composed of multiple RFs to predict the potential miRNA–disease associations. To confirm the excellence of the RF-based ensemble learning model, we compare it with several common classifier models, such as SVM, k-nearest neighbor (KNN), and decision tree (DT), using a common data set and feature set. Figure 5 is the ROC curve of these four classifier models, where the AUC of SVM, KNN, DT, and PMDFI are 0.9336, 0.8348, 0.9171, and 0.9404, respectively. From the picture, the performance of SVM is slightly worse than PMDFI; the AUC of DT is 2.33% lower than PMDFI; the performance of KNN is the worst among them, and its AUC is 10.56% lower than PMDFI. In summary, our method, RF-based PMDFI, has a curve above all the other three ones, which stands for the best performance in predicting miRNA–disease associations.

### 3.6. Analysis of High-Order Feature Extraction and Feature Interaction

**Figure 6:**
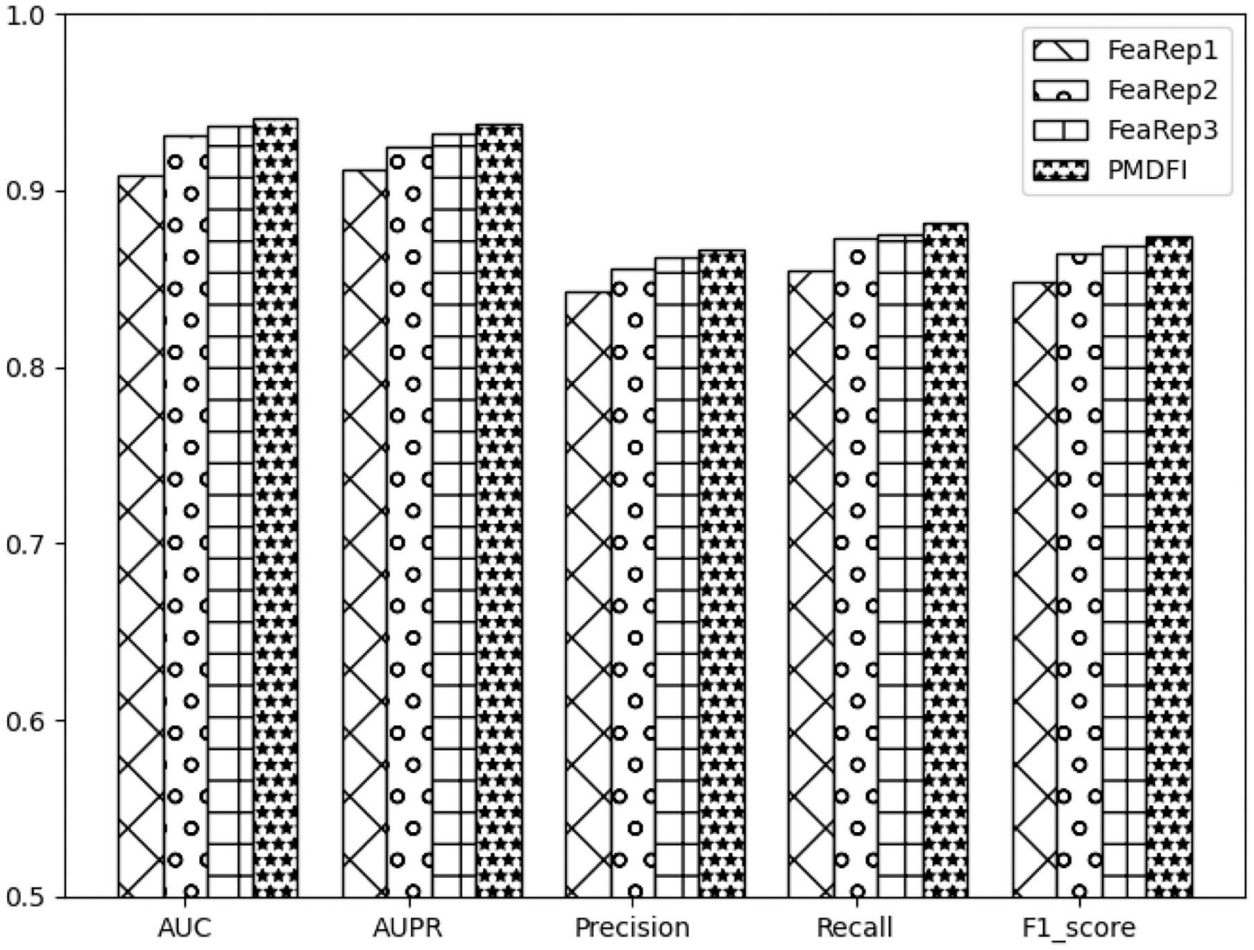
Histograms of comparison of performance based on different feature representation methods.

Unlike other models that directly use miRNA and disease similarity feature information, our method PMDFI utilizes high-order feature extraction and feature interaction to represent features. In order to verify the validity of the proposed feature representation approach, we compare it with other three methods. The first one is DBNMDA (Chen et al., 2020), which directly extracts the features of all miRNA–disease pairs to pre-train the Restricted Boltzmann Machine (RBM). The second one is DBMDA (Zheng et al., 2020), which utilizes the autoencoder to resize the miRNA (disease) similarity features and then fuses the features during the feature set construction stage. The third one is GBDT-LR (Zhou et al., 2020), which uses gradient boosting decision tree (GBDT) to extract distinguishing features and feature combinations. We name the feature representation in each of the aforementioned three methods as FeaRep1 (based on DBNMDA), FeaRep2 (based on DBMDA), and FeaRep3 (based on GBDT-LR). Table 4 reveals in details the outcome of distinct feature representation methods. The AUC of the feature representation method used in the PMDFI are 3.21, 0.97, and 0.37% higher than FeaRep1, FeaRep2, and FeaRep3, respectively. And we plot more straightforward histograms to illustrate the results of the comparison, as shown in Figure 6. From the figure, the feature representation method used by PMDFI, the rightmost one, is superior to the other three methods in all evaluation dimensions. To summarize, the experiment further demonstrates that high-order feature extraction and feature interaction have profound contributions to predicting the potential relevance of miRNA–disease.

**Table 4:** The specific outcomes based on different feature representation methods.

**Method**	**AUC**	**AUPR**	**Precision**	**Recall**	**F1-score**
FeaRep1	0.9083	0.9119	0.8430	0.8543	0.8486
FeaRep2	0.9307	0.9252	0.8554	0.8731	0.8641
FeaRep3	0.9367	0.9327	0.8619	0.8746	0.8682
PMDFI	0.9404	0.9373	0.8663	0.8812	0.8736

### 3.7. Case Studies

To analyze the prediction performance of PMDFI in practical situations, we conduct several common disease case studies with PMDFI, including breast cancer, melanoma, and lymphoma. We initially train all known miRNA–disease associations in the HMDD v.2.0 with PMDFI, and then list top-10 predicted miRNAs for validation using two other databases, namely dbDEMC 2.0 (Yang et al., 2017) and miRCancer (Xie et al., 2013). The dbDEMC 2.0 is a database designed to store and display differentially expressed miRNAs in detected human cancers, which contains 2,224 differentially expressed miRNAs in 36 cancer types. And the miRCancer is a microRNA–cancer association database, which currently records 878 relationships between 236 miRNAs and 79 human cancers.

According to recent studies, we choose three prevalent diseases as our case studies and the results are listed in Table 5. The first one is breast cancer, as the most common cancer affecting women, which accounts for 23% of all cancers and 14% of cancer deaths (Jemal et al., 2011; Anastasiadi et al., 2017). The studies have shown that loss of the tumor suppressor miRNA or overexpression of the oncogenic miRNA may lead to the occurrence or metastasis of breast cancer (Serpico et al., 2014). Therefore, finding the relationship between miRNAs and breast cancer offers a direction for the diagnosis and treatment of breast cancer. From Table 5, we can see that nine out of the 10 predicted breast cancer related miRNAs appear in dbDEMC 2.0 or miRCancer. The second disease is Melanoma, which is the most serious type of skin cancer. It is caused by the cancerous transformation of skin cells when prolonged exposing under the ultraviolet light (Rastrelli et al., 2014). Pencheva et al. (2012) have identified a set of miRNAs that are deregulated in independent metastatic lines derived from multiple patients with melanoma, which manifests the importance to research the association between miRNAs and melanoma. The data from the middle line of Table 5 illustrate that the PMDFI model has accurately predict all the top 10 melanoma-related miRNAs. The last disorder is malignant lymphoma, which is a large group of tumors with considerable heterogeneity. Although it occurs in the lymph nodes, due to the distribution characteristics of the lymphatic system, lymphoma is a systemic disease that can invade almost any tissue and organ in the body (Dean et al., 2005; Paydas et al., 2016). Zheng et al. (2018) list several examples to describe miRNAs' role in the development of B-cell lymphoma, both as oncogenes and tumor suppressor genes, and nine out of the 10 predicted lymphoma-associated miRNAs are verified in dbDEMC 2.0 or miRCancer.

**Table 5:** The candidate miRNAs associated with breast cancer, melanoma, and lymphoma.

**Diseases**	**miRNA**	**Evidence**
	hsa-mir-150	dbDEMC 2.0;miRCancer
	hsa-mir-15b	dbDEMC 2.0
	hsa-mir-130a	dbDEMC 2.0;miRCancer
	hsa-mir-196b	dbDEMC 2.0
Breast cancer	hsa-mir-98	dbDEMC 2.0;miRCancer
	hsa-mir-106a	dbDEMC 2.0;miRCancer
	hsa-mir-142	miRCancer
	hsa-mir-378a	Unconfirmed
	hsa-mir-30e	miRCancer
	hsa-mir-372	dbDEMC 2.0;miRCancer
	hsa-mir-150	miRCancer
	hsa-mir-373	miRCancer
	hsa-mir-127	dbDEMC 2.0
	hsa-mir-181b	dbDEMC 2.0
Melanoma	hsa-mir-10b	dbDEMC 2.0;miRCancer
	hsa-mir-224	dbDEMC 2.0;miRCancer
	hsa-mir-101	dbDEMC 2.0;miRCancer
	hsa-mir-223	dbDEMC 2.0
	hsa-mir-27a	dbDEMC 2.0;miRCancer
	hsa-mir-30c	dbDEMC 2.0
	hsa-mir-34a	dbDEMC 2.0;miRCancer
	hsa-mir-34c	Unconfirmed
	hsa-mir-9	dbDEMC 2.0;miRCancer
	hsa-mir-29a	dbDEMC 2.0;miRCancer
Lymphoma	hsa-mir-222	dbDEMC 2.0
	hsa-mir-7a	dbDEMC 2.0
	hsa-mir-29b	dbDEMC 2.0;miRCancer
	hsa-mir-181b	dbDEMC 2.0
	hsa-mir-145	dbDEMC 2.0;miRCancer
	hsa-mir-221	dbDEMC 2.0

## 4. Conclusion

Given the significance that the miRNA–diseases associations make to the diagnosis of diseases and superiority that computer have compared to biological experiments, emerging computational models pop up in the miRNA–disease associations prediction realm. In this paper, we propose a novel computational model called PMDFI, which is an ensemble learning method to predict the miRNA–disease associations based on feature interactive learning. Our method not only integrates the four RF models of separated cross features, but also incorporates logistic regression to provide comprehensive predictions by assigning adjustable weights. Moreover, we apply stacked autoencoders to extracting meaningful high-order features from miRNA functional similarity, disease semantic similarity, and GIP kernel similarity of miRNA and disease. And we also construct a feature interaction layer to promote the interactions between distinct features. As a result, PMDFI reaches the average AUC of 0.9404 and 0.9415 under 5-fold and 10-fold cross-validation and successfully predicted miRNA–disease associations within three case studies.

However, there is room for improvement in the future. First, with the rapid development of sequencing technology, all types of data have exploded, and we will integrate those multi-source data to dramatically improve the robustness of the model. Second, in future researches, we would devote ourselves to discovering more original features of miRNAs and diseases to boost the performance and explore some brand-new feature calculation methods. Third, concerning the negative samples, we randomly select them from unlabeled samples, which may include unreliable false samples. To offset these negative effect on the eventual prediction, we would introduce the measurement of reliable negative samples in the future.

## Data Availability Statement

The datasets presented in this study can be found in online repositories. The names of the repository/repositories and accession number(s) can be found in the article/supplementary material.

## Author Contributions

LD, MT, and JiL conceived the prediction method. MT and JuL wrote the paper. MT, CL, and DL developed the computer programs. CL and DL analyzed the results and revised the paper. All authors contributed to the article and approved the submitted version.

## Conflict of Interest

The authors declare that the research was conducted in the absence of any commercial or financial relationships that could be construed as a potential conflict of interest.
